# Maternal Responsiveness to Infant Distress: A Cross-Cultural Investigation in Uganda and the United Kingdom

**DOI:** 10.1037/dev0002038

**Published:** 2025-09-04

**Authors:** Carlo Vreden, Santa Atim, Joanna C. Buryn-Weitzel, Giammarco Di Gregorio, Ed Donnellan, Maggie Hoffman, Eve Holden, Michael Jurua, Charlotte V. Knapper, Sophie Marshall, Josephine Paricia, Florence Tusiime, Claudia Wilke, Katie E. Slocombe, Zanna Clay

**Affiliations:** 1 Department of Psychology, Durham University; 2 DIPF|Leibniz Institute for Research and Information in Education, Frankfurt am Main, Germany; 3 Budongo Conservation Research Station, Masindi, Uganda; 4 Department of Psychology, University of York; 5 Department of Psychology, University of Warwick; 6 School of Human Evolution and Social Change, Arizona State University

**Keywords:** maternal responsiveness, parenting practices, emotion regulation, cross-cultural

## Abstract

Maternal responses are a key factor in shaping early emotional development. However, research on how mothers respond to infant emotional signals outside of Western industrialized contexts remains limited. This study provides a longitudinal, naturalistic approach to mapping cultural variation in maternal responsiveness and its effect on infant emotional outcomes. To do so, we used naturalistic video observations to assess spontaneous maternal responses to infant distress and their link with infant recovery from distress in a cross-cultural sample. Data were collected on 82 mother–infant dyads (46 female) at 3 and 6 months old, from two distinct cultural settings: Uganda and the United Kingdom. Although maternal responses were faster in the United Kingdom, infant recovery was quicker in Uganda, suggesting that culturally specific maternal strategies of responding may be more effective than simply promptness of responses in reducing distress. Further, we found changes in maternal response strategies by age and that some of these differed by site. Our findings show both cross-cultural continuity and variability in maternal responses to infant distress and broaden our understanding of how early infant–caregiver interactions shape early socioemotional development.

## Public Significance Statement

Our study highlights how mothers in different cultural contexts respond to infant distress and how these responses shape infants’ emotional development. By comparing naturalistic mother–infant interactions in Uganda and the United Kingdom, our research reveals that culturally specific caregiving strategies—not just how quickly mothers respond—play an important role in helping infants recover from distress.

Throughout daily interactions, infants’ primary caregivers play a critical role in shaping early socioemotional development. By responding to and helping to regulate their child’s emotions, caregivers shape how children understand and regulate their own emotional states and eventually respond to others’ emotions (Eisenberg et al., 2010; Gergely & Watson, 1999; Lavelli et al., 2019). Research has shown that variation in caregiver responsiveness to children’s emotional and communicative signals affects a range of developmental outcomes, including socioemotional, communicative, and cognitive development (Deans, 2020; Landry et al., 2006). However, much of this research has focused on populations in the Global North, despite these not being representative of the majority of the world’s population. For example, in a recent meta-analysis on sensitive caregiving and attachment outcomes, only 2% of studies included samples from Africa and South America (Madigan et al., 2024). Expanding our understanding of caregiving strategies across diverse cultures is essential to fully understand their culture-specific effects on child outcomes.

Infant distress vocalizations, such as crying, elicit caregiving behaviors in a wide variety of species, suggesting ancient evolutionary roots (Bowlby, 1982; Lingle et al., 2012). In humans, infants across diverse settings, including those from France, Japan, Fiji, Kenya, and the United States, emit comparable rates of distress vocalizations (Bornstein et al., 1992; Broesch et al., 2016). While caregivers consistently attend to infant distress, their responses—especially when there is no immediate threat to the infant—vary across cultures in terms of both speed and style. For instance, in their infants’ first 3 months of life, German and Italian mothers primarily employed verbal responses, while Nso mothers from rural Cameroon used more tactile behaviors (Kärtner et al., 2010; Lavelli et al., 2019). Response modality can vary not only with cultural setting but also with infant age. Moreover, differences in maternal response styles between Gusii mothers from rural Kenya and American mothers were found to be smaller at 3–4 months than at 9–10 months (Richman et al., 1992). With younger infants, the most frequent maternal behavior in both cultural contexts was to hold the infant. However, with increasing age, American mothers most frequently looked at or talked to their infant, while Gusii mothers’ preferred response remained holding the infant. This suggests that cultural variation in maternal response strategies may become more pronounced as infants develop, highlighting the importance of considering the interaction between age and culture when examining differences in maternal responsiveness.

Cultural differences not only influence how mothers respond to infant emotions but also shape their interpretations of the causes of infant distress, which in turn affects their caregiving responses. For instance, Gamo mothers in rural Ethiopia interpreted infant distress as a sign of possible illness, believing that breastfeeding and physical contact were the most appropriate interventions (Bader & Fouts, 2018). In settings where infant mortality is high, such as rural Ethiopia, this focus on physical causes of distress may reflect the urgent need for intervention (LeVine, 1980). Conversely, in sociocultural contexts with lower risks to infant health, where emotional autonomy is emphasized, caregivers may interpret infant distress as an emotional expression rather than a physical need. These caregiving prototypes—one Western, focused on emotional autonomy and verbal responses, and one non-Western, relational, emphasizing physical caregiving—provide a useful framework for studying maternal responsiveness across cultures, though significant within-culture variation is to be expected (Keller & Otto, 2009).

Maternal responses to infant distress not only help to regulate the infant’s emotions in the moment but also contribute to their long-term capacity for emotion regulation (Cole et al., 2004). Initially, caregivers are said to regulate infant emotions through a kind of bio-feedback loop, supporting the infant’s organization of emotional experiences (Gergely & Watson, 1999; Halberstadt & Lozada, 2011; Stern, 1985). Over time, through receiving comfort in response to their internal experiences, external regulation provided by the caregiver is gradually transferred to the infant, who develops independent emotional regulation abilities (Calkins & Hill, 2007). Supporting this process, research has found that infants from Italy and the United Kingdom who received maternal responses to a greater proportion of their emotional signals in the first year of life demonstrated more mature emotion regulation by age 2 (Bozicevic et al., 2021). However, some studies suggest that a lack of maternal response to distress may also promote emotion regulation. For instance, Dutch infants whose mothers did not actively respond to mild distress cried less frequently over time, a phenomenon the authors referred to as “benign neglect” (Van Ijzendoorn & Hubbard, 2000).

It should be noted that maternal responsiveness, that is, the promptness and appropriateness of a mother’s response to her child’s communicative signals, may operate differently in distressing versus nondistressing contexts and may have different effects on infant socioemotional outcomes. Maternal responsiveness to infant distress (e.g., soothing a crying infant) but not to nondistress is linked with fewer behavioral problems and higher social competence in early childhood (Leerkes et al., 2009). Responsiveness to nondistress vocalizations, on the other hand, is linked with infant language ability (Tamis-LeMonda et al., 1996). This distinction may be particularly relevant in cross-cultural research: Sensitivity to distress cues may be a more universal caregiving behavior, given its evolutionary function of addressing causes for distress to ensure infant survival, while responsiveness in nondistress situations may often vary across cultures, possibly due to differing parental expectations regarding autonomy, social engagement, and the role of caregiver intervention.

Cross-cultural research on maternal responsiveness and child emotional development is limited. One study found that 2-year-olds in an urban U.K. setting and two South African settings—one White, Afrikaans, urban group and one Black, isiXhosa, peri-urban group—responded differently to frustration depending on the responsiveness their mothers had shown when the infants had been 3 months old (Bozicevic et al., 2016): In U.K. infants, higher maternal sensitivity to infant nondistress emotional signals (e.g., smiling) was linked with more use of distraction by the infant, but in the peri-urban South African sample, the opposite was the case. Additionally, maternal dismissal of infant distress, frequent in the peri-urban isiXhosa group, was associated with more passive responses of the infant to frustration, while acknowledgment of distress, more common in the urban Afrikaans group, led to more active emotion regulation strategies. This variability emphasizes the differential effects of maternal responses to infant distress and nondistress signals and the importance of distinguishing clearly between the two in their assessment.

Taken together, the findings suggest that the relationship between maternal responses to infant emotions and their link with emotion regulation is complex and warrants culturally sensitive reexamination. As part of this reexamination, it is important to acknowledge that the appropriateness of applying Western concepts, such as sensitivity or responsiveness, to non-Western contexts remains debated (Keller et al., 2018; Mesman, 2018; Mesman et al., 2018). These terms refer to the prompt and appropriate response of a caregiver to an infant’s emotional signals; nevertheless, the judgment of what is appropriate can be subjective and, at present, is also heavily biased toward Western ideals of “good” caregiving practices. For example, while Western frameworks of responsiveness (to distress and nondistress) typically emphasize contingent verbal engagement and emotional expressiveness between mother and infant, many non-Western caregiving systems prioritize physically close, anticipatory care and more muted emotional expressivity. For example, in Cameroonian Nso communities, maternal responsiveness is frequently expressed through physical closeness but also through commands to the infant not to express negative affect (Keller et al., 2018). On one hand, these strategies can be seen as appropriately preparing the infant to fit into their cultural context; on the other hand, if only assessed through a Western cultural lens, they could also be assessed negatively. This contrast illustrates how the concept of responsiveness is strongly embedded in broader cultural models of parenting, underscoring the need for culturally sensitive assessments of maternal behaviors. What is considered responsive in one culture may not be regarded as appropriate or lead to the same developmental outcomes in another.

Taking this into account, the present study aims to expand our understanding of cross-cultural variation in maternal responses to infant distress and to naturalistically examine how these responses may influence child outcomes during the first year of life. We use the term “maternal responsiveness” to describe the ways in which mothers respond to their child’s signals, focusing on the type and promptness of responses. Using naturalistic video observations, we analyze the speed and style of maternal responses to infant distress at two time points (3 and 6 months) and assess their impact on infant recovery from distress—a behavioral proxy for emotion regulation. Owing to its observational design, our naturalistic data preclude identification of the cause of distress in each episode, which is needed to assess maternal responses appropriateness and thus we did not evaluate this aspect. This approach moreover ensures we avoid value judgments about their appropriateness across different cultural contexts. Our cross-cultural, longitudinal sample includes mothers and infants from two distinct settings: the United Kingdom and Uganda. The U.K. sample was from an industrialized, semiurban setting with small nuclear family households, engagement in wage labor, and high maternal education. The Ugandan sample was from a subsistence farming community where most families live in multigenerational large households, formal employment is rare, and maternal education is low. Previous research with these populations revealed differences in caregiving practices, with Ugandan infants experiencing more physical contact with caregivers and receiving care from more different caregivers compared to U.K. infants (Holden et al., 2022).

Our main prediction was that U.K. and Ugandan mothers would vary in their latency and style of responses to infant distress. However, we did not make directional predictions given that our analysis is exploratory, and multiple factors may be at play. In one regard, previous research conducted on the samples used in the present study has shown that the Ugandan mothers value relational socialization goals (Holden et al., 2022), which have been linked with a preference for less emotionally expressive infants (Keller & Otto, 2009). Socialization goals refer to the long-term developmental priorities of caregivers, which shape caregiving practices and expectations and are influenced by the caregiver’s sociocultural background (Keller, 2007). This could therefore result in Ugandan mothers providing faster maternal responses compared to U.K. mothers, to help the infant return to a state of emotional neutrality as quickly as possible. Alternatively, the Ugandan mothers were from subsistence farming settings, where work demands on women tend to be high (LeVine, 1998). Assuming the child is safe, this may instead make a later response or no active response more favorable, to avoid interrupting the physical work a mother is currently engaged in.

In term of age, we expected mothers from both Uganda and the United Kingdom to respond faster when their child was 3 months than 6 months, given their greater vulnerability and dependency. In addition, emotion regulation is proposed to be gradually transferred from the caregiver to the infant through coregulating interactions (Gergely & Watson, 1999); thus, constant immediate intervention might be less necessary. However, it is possible that this increase of maternal response latency with age might interact with sociocultural setting, owing to different cultural values surrounding expression and management of children’s emotions (e.g., Keller & Otto, 2009).

Second, we tested whether maternal response latency to infant distress influenced infant behavioral recovery from distress. Given the limited number of non-Western findings on this link and the mixed results within Western contexts on whether infant emotion regulation is supported by prompt maternal responses (Bozicevic et al., 2016, 2021) or a lack thereof (Van Ijzendoorn & Hubbard, 2000), this analysis of the association between latency of maternal responses and infant recovery and how these differed across cultures was exploratory.

Third, we examined maternal soothing strategies and whether these varied with infant age and sociocultural setting. Given that infants from the Ugandan site experience more body contact with their caregivers across behavioral contexts (Holden et al., 2022), we expected more physical strategies (increased contact and breastfeeding) primarily aimed at quickly recovering a neutral emotional state that are proposed to be typical for rural African settings (Kärtner et al., 2010; Keller & Otto, 2009). By contrast, we expected strategies allowing more emotional expression in the United Kingdom, such as verbally questioning the infant’s needs or reasons for distress, and reduced reliance on physical comforting. We took an exploratory approach to assessing how any cross-cultural variation in maternal response behaviors might change with age.

In sum, maternal responsiveness plays a significant role in shaping infant emotion regulation, though its effects may vary across cultural settings. This study seeks to map out culturally specific caregiving practices and their links to developmental outcomes, thereby contributing to a more nuanced understanding of early socioemotional development across diverse cultures.

## Method

### Transparency and Openness

We report all data exclusions and measures used in this study. All statistical analyses were carried out in R (R Core Team, 2020). All data and analysis code are available at https://osf.io/w74dm/. This study was not preregistered.

### Participants and Research Sites

In total, 82 infants and their mothers participated in this study. The present study was part of a larger longitudinal project following these mother–infant dyads over the first 2 years of life. See Table 1 for full participant information. A more detailed description of the demographic characteristics of the samples is available in Holden et al. (2022).[Table tbl1]

#### Uganda

The Ugandan sample lived in villages in Masindi district in Western Uganda. Participating families were mostly subsistence farmers living in large multigenerational households, with limited access to electricity, Western media, and formal education. Although mothers are still the primary caregiver for infants at 3–6 months old, they often receive help from others in the household or the village. Mothers were recruited by word of mouth and at village or church meetings by local research assistants from the community. Initial screening of available videos resulted in 101 distress events at 3 and 194 distress events at 6 months from the Ugandan sample.

#### The United Kingdom

The U.K. sample lived in and around the city of York in the United Kingdom. Participating families had access to secondary education and beyond and engaged in wage labor (when not on maternity leave). U.K. mothers were recruited at children’s centers and baby classes and through social media. Initial screening of available videos resulted in 172 distress events at 3 and 140 distress events at 6 months from the U.K. sample.

### Ethics

Ethical approvals were obtained from the Department of Psychology Ethics Committees at the University of York and Durham University, the Ugandan Virus Research Institute Regional Ethics Committee, and the Ugandan National Council for Science and Technology (Reference: SS4545). All mothers provided consent for their and their child’s participation. In the United Kingdom, mothers gave written consent. In Uganda, due to the low literacy levels in our sample, local research assistants, fluent in English and the local languages mothers were fluent in, read out the information sheet, and mothers consented verbally and with a thumb print in lieu of a signature.

### Procedure

#### Data Collection

Video observations of naturally occurring social interactions among mother–infant dyads were collected during home visits when infants were 3 and 6 months old. In Uganda, a local research assistant fluent in the mother’s language visited the family’s home to conduct naturalistic behavioral observations throughout the day, usually for 8 hr (approx. 9 a.m.–5 p.m.). In the United Kingdom, observational videos were collected by local research assistants during the 2-hr home visits.

During the home visit, the researcher in both settings opportunistically filmed everyday scenarios involving the infant, such as bathing, feeding, playing, and resting. To promote ecological validity, we filmed in the most typical location where the mother and infant could be expected to spend most of their day. In Uganda, most videos were therefore filmed outside in the family’s compound, in the yard between buildings, but some were recorded inside the home. In the United Kingdom, all videos were collected in the home. All video observations were recorded on Panasonic high-definition camcorders (HC-VX870 4K and HC-V high-definition) with external microphones (Sennheiser MKE 400 shotgun microphone).

#### Video Coding

All coding was completed using ELAN, an open-source audio and video annotation software (Wittenburg et al., 2006). A total of 839 video observations (ranging in length from ca. 2–20 min) were collected across the two time points and sites. These were screened for all occurrence of infant distress episodes, which occurred in 147 videos, with each infant contributing at least one distress episode. Only episodes of detectable vocal distress lasting a minimum of 2 s (e.g., audible whimpering, crying) were coded. Distress facial expressions occurring in the absence of vocalizations were not included owing to challenges in reliable detection (videos were taken from a single angle; thus, facial expressions were not always visible). Additionally, focusing on vocal distress allowed us to include naturalistic scenarios in which mothers were engaged in other tasks and not attending to their child visually, as opposed to restrained, seated interaction scenarios which may have provided better visibility.

Distress signals were coded into two intensity categories, mild and high, to control for potential differences in maternal response latency or strategies for different levels of distress. Mild distress signals included mild and/or intermittent whimpering or fussing (a series of low, feeble sounds) or a single cry sound. High distress signals included crying or sobbing (i.e., continuous, rhythmic distress vocalizations) and/or high-pitched shrieking. Only distress events of 2 s or more were coded, and events were considered separate events if they were separated by 5 s or more of no vocalization. We applied this time criterion in order to avoid subjective judgments of which episodes were caused by the same initial distress and which were causally separate. This resulted in 607 individual distress events. Most infants contributed data from more than one distress episode. Supplemental Table S2 contains information about the mean number and range of distress episodes contributed per infant (by site and age). Although we were interested in potential causes of distress, we did not code this from the videos, after an initial screening revealed that this was difficult to reliably ascertain, as most distress events occurred without an obvious cause.

We also coded the type of maternal response to infant distress, from the onset of infant distress until offset of infant distress. Six active maternal responses included (a) increases in physical contact with the infant (e.g., picking them up or holding the infant closer if already touching), (b) engaging the infant’s attention without changes in physical contact (e.g., snapping fingers), (c) breastfeeding, (d) other feeding (bottle, solids), and (e) verbal or (f) nonverbal sounds directed toward the infant. Finally, mothers could (g) show no active response to their infant’s distress (this does not preclude attention to the distress; rather, it describes no change in maternal behavior toward the infant beyond visual attention). We chose these response categories after pilot coding based on patterns which would be applicable to both cultural contexts. Distinction between verbal and nonverbal vocalizations in Uganda was based on written translations of all videos by research assistants fluent in English and the local languages. All active responses were not mutually exclusive and could overlap. Other (nonbreast) feeding was not further analyzed due to very low frequencies of occurrence. See Supplemental Text S1 for the full coding scheme.

Last, to control for proximity, we coded the distance between the mother and her child at distress onset. We categorized mother–infant distance into five categories, (a) already in physical contact, (b) infant within (maternal) arm’s reach (i.e., the mother could touch them by extending her arms but not moving the rest of her body), (c) infant within three adult steps, and (d) infant further than three adult steps away. If only the infant but not the mother was visible in the video ([e] out of shot) and it was thus unclear if and how soon the mother could hear the infant’s distress vocalization, this distress event was not considered for further analysis. These distance measures could be reliably identified in the videos, see below for intercoder reliability.

From our coding, we then extracted timestamps for the on- and offset of infant distress and onset timestamps of active maternal responses. Distress events which triggered an active maternal response were used to calculate maternal response latency (the time elapsed between infant distress onset and the first active maternal response onset) and, for distress events with a maternal response, infant recovery from distress (the time elapsed between the onset of the first active maternal response and the offset of infant distress). Although distress duration, based on the time elapsed between the on- and offset of infant distress, was not analyzed separately, the mean duration of distress episodes per site and infant age can be found in the Supplemental Text S2.

A second coder coded 20% of the video observations for distress on- and offsets, response onsets, categorization of response types, maternal distance from the infant, and distress intensity. Intraclass correlations for timestamps were high (0.99–0.99), as were Cohen’s kappas for the different maternal response type categories, the mothers’ distance from the infant at distress onset, and for distress intensity (к = .86–.94), indicating good reliability.

### Statistical Analyses

Statistical analyses were carried out in R Core Team, 2020). We used the package lme4 (Bates et al., 2015) for linear mixed effect models and generalized linear mixed models and the drop1 function to extract *p* values for individual model terms. We compared all full models to null models (that consisted of just the control variables and random effects), using likelihood-ratio tests (Dobson, 2002), to establish overall model significance. For models containing interaction terms, if the full-null model comparison was significant, we compared the full model (with the interaction) to a reduced model (without the interaction term), also using likelihood-ratio tests. We considered a full model to be a significantly better fit than a reduced model if the likelihood-ratio test produced a *p* value < .05; otherwise, we continued with the reduced model. Model estimates reported in the Supplemental Text S2 are for full models, unless the full-reduced model comparison was not significant, in which case we report the reduced model (excluding the interaction term). All models met assumptions of no collinearity, checked using variance inflation factors (Field, 2005). Nonbinomial generalized linear mixed models showed no overdispersion, checked using a function provided by Roger Mundry (personal communication). We assessed model stability by using the function glmm_stability and found all our models to be stable. Post hoc contrasts were tested using the emmeans package (Length, 2022). In total we ran eight different models to answer our research questions (see Table 2 for an overview of model structure, including outcome and predictor variables). All models included a random intercept of infant participant ID to account for repeated contributions. Data and analysis code are available at https://osf.io/w74dm/. This study and the analyses were not preregistered.[Table tbl2]

## Results

### Latency of Maternal Response to Infant Distress

The full model was significantly better at explaining variation in maternal latency to respond to infant distress compared to a null model, χ^2^(3) = 17.37, *p* < .001. However, the full model, which contained the interaction between site and age, was not significantly better than the reduced model, which contained the same main and random effects without the interaction, χ^2^(1) = 1.91, *p* = .168. Therefore, we performed single term deletions with the reduced model to ascertain the main effects of site and age on maternal response latency. Mothers from the United Kingdom tended to respond faster than Ugandan mothers (estimate ± *SE* = −0.27 ± 0.13, χ^2^(1) = 4.45, *p* = .035; see Figure 1). Mothers from both sites were faster to respond when their child was 3 months old compared to 6 months (estimate ± *SE* = −0.39 ± 0.13, χ^2^(1) = 9.33, *p* = .002; see Figure 2). See Supplemental Text S2 for model estimates, standard errors, and confidence intervals for all models.[Fig fig1][Fig fig2]

### Relationship Between Latency of Maternal Response and Infant Recovery

The full model was significantly better at explaining variation in infant recovery from distress compared to a null model, χ^2^(4) = 12.92, *p* = .012. However, the full model, which contained the interaction between site and maternal response latency, was not significantly better than the reduced model, which contained the same main and random effects without the interaction, χ^2^(1) = 0.37, *p* = .543. Therefore, we performed single term deletions with the reduced model to ascertain the main effects of site and latency on infants’ recovery time. Longer maternal response latencies were associated with slower infant recovery from distress (estimate ± *SE* = 0.11 ± 0.05, χ^2^(1) = 5.15, *p* = .023; see Figure 3). Additionally, infants from the U.K. sample took longer to recover from distress than Ugandan infants, regardless of maternal latency to respond (estimate ± *SE* = 0.30 ± 0.12, χ^2^(1) = 6.23, *p* = .013; see Figure 4). There was no effect of age on infant recovery from distress (estimate ± *SE* = −0.17 ± 0.12, χ^2^(1) = 1.95, *p* = .162).[Fig fig3][Fig fig4]

### Maternal Response Strategies

#### Increasing Contact

The full-null model comparison was not significant, χ^2^(3) = 4.31, *p* = .230, indicating that, contrary to our prediction, there was no effect of cultural setting or infant age on maternal tendency to increase physical contact when responding to infant distress.

#### Breastfeeding

The full model was significantly better at explaining variation in mothers’ likelihood to use breastfeeding as a response strategy compared to a null model, χ^2^(3) = 13.01, *p* = .005. The full model explained significantly more variation than the reduced model lacking the interaction term, χ^2^(1) = 4.05, *p* = .044, showing there was a significant interaction between site and infant age on the use of breastfeeding (Figure 5). Follow-up contrasts showed U.K. mothers were more likely to breastfeed their distressed infant at 3 months than at 6 months (estimate ± *SE* = 1.74 ± 0.71, *z* = 2.46, *p* = .014), whereas there was no age-related change for Ugandan mothers (estimate ± *SE* = 0.10 ± 0.47, *z* = 0.20, *p* = .840). At 6 months, Ugandan mothers were more likely than U.K. mothers to use breastfeeding as a strategy when responding to their distressed infant (estimate ± *SE* = 2.15 ± 0.72, *z* = 2.99, *p* = .003), but not at 3 months (estimate ± *SE* = 0.50 ± 0.51, *z* = 0.98, *p* = .327).[Fig fig5]

#### Engaging Attention

The full model was significantly better at explaining variation in likelihood to engage infant attention as a response strategy compared to a null model, χ^2^(3) = 13.73, *p* = .033. The full model explained significantly more variation than the reduced model lacking the interaction term, χ^2^(1) = 4.45, *p* = .035, showing there was a significant interaction between site and infant age on maternal nonphysical engagement of their child’s attention. Follow-up contrasts showed that Ugandan mothers were more likely to engage their child’s attention at 6 than at 3 months (estimate ± *SE* = 1.97 ± 0.78, *z* = 2.58, *p* = .012; see Figure 6). There was no such change in the United Kingdom (estimate ± *SE* = 0.03 ± 0.58, *z* = 0.05, *p* = .959; see Figure 6), nor across sites at either 3 months (estimate ± *SE* = −0.69 ± 0.84, *z* = −0.81, *p* = .416) or 6 months (estimate ± *SE* = 1.26 ± 0.67, *z* = 1.87, *p* = .061).[Fig fig6]

#### Verbal Responses

The full model was significantly better at explaining variation in maternal likelihood to speak to the infant compared to a null model, χ^2^(3) = 32.57, *p* < .001. However, the full model, which contained the interaction between site and infant age, was not significantly better than the reduced model, which contained the same main and random effects without the interaction, χ^2^(1) = 0.02, *p* = .900. Therefore, we performed single term deletions with the reduced model to ascertain the main effects. U.K. mothers were more likely than Ugandan mothers to speak to their infant following distress (estimate ± *SE* = 1.84 ± 0.35, χ^2^(1) = 28.57, *p* < .001; see Figure 7); however, this was not influenced by infant age (estimate ± *SE* = −0.54 ± 0.29, χ^2^(1) = 3.35, *p* = .067; see Figure 7).[Fig fig7]

#### Vocalizations

The full-null model comparison was not significant, χ^2^(3) = 5.14, *p* = .162, indicating that there was no effect of site nor infant age on maternal tendency to use nonspeech vocalizations when responding to their child’s distress.

#### No Active Response to Distress

The full model was significantly better at explaining variation in likelihood to not provide an active response to distress signals as compared to a null model, χ^2^(3) = 19.81, *p* < .001. However, the full model, which contained the interaction between site and age, was not significantly better than the reduced model, χ^2^(1) = 0.15, *p* = .700. Therefore, we performed single term deletions with the reduced model to ascertain the main effects of site and age on mothers’ likelihood to not actively react to infant distress. Mothers from Uganda were more likely than U.K. mothers to not actively respond to distress (estimate ± *SE* = 0.66 ± 0.28, χ^2^(1) = 5.21, *p* = .022; see Figure 8). Mothers from both sites were more likely to not actively respond to distress when their child was 6 months old compared to 3 months (estimate ± *SE* = 0.85 ± 0.25, χ^2^(1) = 13.05, *p* < .001; see Figure 9).[Fig fig8][Fig fig9]

## Discussion

Maternal sensitivity to children’s physical and emotional needs is considered a key factor in shaping early socioemotional development (Ainsworth, 1967; Gergely & Watson, 1999). However, most research has still been primarily conducted in Western contexts, despite evidence that maternal regulation of infant distress varies cross-culturally (e.g., Kärtner et al., 2010; Keller & Otto, 2009; Richman et al., 1992). The present study investigated cross-cultural variation in maternal responses to infant distress and the potential influence of these responses on infant recovery from distress at two time points (3 and 6 months of age) in the United Kingdom and Uganda. We explored potential variation in maternal response latency, strategies employed to soothe infants, and the link between maternal responsiveness and infants’ ability to recover from distress. Our findings, which showed both similarities and differences between the two sites, reveal key insights into how sociocultural contexts shape maternal caregiving practices which may influence infant emotional development.

First, we examined maternal response latency from onset of the distress of the infant to when the mother initiated her first soothing response. U.K. mothers responded faster to infant distress than Ugandan mothers, suggesting that response speed is not necessarily associated with the cultural value placed on emotional neutrality, as previously hypothesized (Keller & Otto, 2009). Instead, U.K. mothers may place a greater emphasis on promptly addressing emotional signals through direct interventions, in line with the highly responsive caregiving style emphasized in Western caregiving (Keller, 2007; Keller & Otto, 2009). In contrast, Ugandan mothers’ slower responses may be shaped by a combination of contextual and cultural factors: One possibility is that the high work demands in subsistence farming settings constrain immediate responding to infant distress, especially when there is no immediate threat to the infant (LeVine, 1998). Another complementary explanation is that Ugandan mothers may delay their responses, as a way of encouraging infant self-regulation, which would also align with socialization goals emphasizing emotional inexpressiveness (Keller & Otto, 2009). Both groups of mothers responded faster when their infants were younger (3 months compared to 6 months), which is consistent with previous findings that highlight the role of early caregiver–infant coregulation (Calkins & Hill, 2007). As infants age, maternal response latency increased: Proximately, this could reflect their perceived growing robustness and independence, while also reflecting a maternal strategy to gradually promote greater infant self-regulation (Calkins & Hill, 2007; Gergely & Watson, 1999). The overall slower latency of responses in Uganda may be a sign that this transfer from coregulation to self-regulation is expected to occur earlier by Ugandan mothers. Future research examining maternal activities, socialization goals for emotion regulation, and their responses to infant signals would add valuable insights into disentangling their overlapping influences.

Across both sites, slower maternal responsiveness was associated with slower infant recovery from distress, suggesting that prompt maternal intervention can facilitate quicker emotional regulation in infants (Bozicevic et al., 2016, 2021). However, despite Ugandan mothers overall responding later to infant distress, Ugandan infants recovered faster from distress. In that sense, Ugandan maternal responses—or at least their effect—appeared to mostly align with the relational socialization goals found in other rural African settings to quickly return infants to an emotionally neutral and socially harmonious state (Keller & Otto, 2009). These findings indicate that while prompt maternal responses may play a role in infant recovery, other factors such as cultural response strategies may also influence recovery time. Given that this study is based on observational data, we can only speculate with regard to the mechanisms which support the infant to regulate their emotions in this way. Unmeasured factors not captured in our behavioral coding, such as infant temperament or the reasons for infant distress, could also impact both maternal behavior and infant distress recovery. Moreover, recovery from distress served as a behavioral proxy for emotion regulation. This measure should be seen as a starting point but does not capture the full range of emotional and cognitive processes involved in regulating emotions. Further research, particularly longitudinal or experimental studies, is needed to clarify these relationships and assess how early maternal responsiveness influences the long-term development of emotion regulation.

Beyond maternal response latency, we also found several differences in maternal response strategies between the two cultural contexts. Ugandan mothers were more likely to use breastfeeding as a soothing strategy at 6 months compared to U.K. breastfeeding mothers, and their likelihood of breastfeeding in response to infant distress did not change with infant age. In other rural African contexts, breastfeeding has been described as the preferred distress response (Bader & Fouts, 2018; Keller & Otto, 2009), which reflects a cultural emphasis on physical contact and bodily care as primary means of infant comfort (Kärtner et al., 2010). U.K. mothers, on the other hand, may prioritize other forms of distress responses, such as verbal responses, as their infants age, fostering emotional autonomy and infant communication abilities. In our sample, all Ugandan mothers and 70% of U.K. mothers (as compared to 81% at 3 months) still breastfeed their child at 6 months. This data suggest that most U.K. mothers were still engaged in breastfeeding at both time points but were less likely to be using it as a soothing strategy. However, due to the decrease in the number of U.K. mothers who still breastfed at this age, a future comparison of tendency to breastfeed in nondistress versus distress situations is needed to rule out the alternative explanation that this change in strategy is simply due to overall reduced breastfeeding rates.

Engagement of infant attention also showed cultural differences, with Ugandan mothers more likely to use this strategy as infants aged, while U.K. mothers exhibited no age-related change. This pattern in Uganda may reflect a growing emphasis on nonphysical strategies as infants become more capable of interacting with their environment, while U.K. mothers may rely more on nonphysical cues from the outset. Verbal responses were more common among U.K. mothers, aligning with research that suggests highly verbal, face-to-face interaction is characteristic of Western, autonomy-oriented caregiving styles (Keller & Otto, 2009).

There were also similarities in maternal strategies between the two samples: Mothers from both sites were equally likely to increase contact with their distressed infant, in contrast with previous research based on which one might expect Ugandan caregiving to be more physical (Richman et al., 1992). This finding underscores the importance of nuanced mappings of cultural variation in caregiving practices, to avoid a fallacy of oversimplifying Western and non-Western caregiving into less and more physical caregiving.

Because our coding of maternal responses was not mutually exclusive and distress episodes often received more than one type of response (such as breastfeeding and talking to the infant at the same time), it did not allow us to make inferences about links between certain maternal behaviors and infant outcomes. Therefore, we cannot point to a specific difference in maternal response strategy that would explain the finding that despite longer latencies, Ugandan infants recovered from distress faster than U.K. infants.

There is one other difference in maternal responses that may have contributed to the difference in infants’ speed of recovery. Both U.K. and Ugandan mothers were less likely to actively respond to distress as their infants aged, which may reflect their perception of their infants’ growing physical robustness as well as expectations that they can start to manage their emotions independently. Although Ugandan infants recovered more quickly than U.K. infants, Ugandan mothers were significantly less likely than U.K. mothers to actively respond to infant distress. Several factors may contribute to this pattern of results. This finding may reflect differences in cultural attitudes toward infant distress, where Ugandan mothers might perceive less need for immediate intervention, possibly due to higher demands on their time as is common in subsistence farming communities or different expectations regarding infant self-regulation (LeVine, 1998). It could also reflect differences in the environment—Ugandan mothers were usually in open compounds where they could visually monitor their infants, and other potential caregivers were often in proximity, reducing potential risks associated with not actively responding immediately. In contrast, U.K. mothers were often the sole caregiver in the house at the time of data collection. Although we do not know the extent to which these potential factors drove this cultural difference, prior research has linked not providing an immediate response to infant distress with better distress regulation (Van Ijzendoorn & Hubbard, 2000). It seems plausible therefore that not always responding immediately to infant distress may thus be effective in this Ugandan context, allowing infants to develop greater independence in managing their emotional states. Previous research has shown that infants whose distress is dismissed by caregivers are more passive when responding to an emotionally arousing scenario, as compared to those whose mothers acknowledge and actively respond to their distress, who later show greater active emotion regulation strategies (Bozicevic et al., 2016). It would be of interest for future research to establish whether the manner in which children in different cultural settings come to regulate their own emotions, shaped through caregiver strategies, is predictive of how they cope with social interactions requiring emotion regulation, such as empathic responding. Understanding how early caregiving experiences build later trajectories of regulating and responding to one’s own and others’ emotions across diverse cultural contexts is critical for building a more inclusive understanding of socioemotional development.

Overall, the findings from this study contribute to a culturally nuanced understanding of maternal responsiveness and its effects on infant development. While overall faster maternal responses facilitated quicker recovery from distress, our findings indicate that the type of maternal response may influence its effectiveness in soothing the infant or supporting emotional regulation. Our study focused on maternal style and response timing of responses, rather than on the response appropriateness in relation to the infant’s specific cues or needs. According to a Western cultural framework, caregiver responses are considered sensitive or appropriate according to their ability to match the underlying cause of distress (e.g., tiredness, hunger, overstimulation). In our purely observational, naturalistic data, we could not reliably identify the cause of distress in each episode, and thus we did not evaluate this aspect. Nevertheless, the link between response latency and recovery from distress provides a first-step measure of response effectiveness. To further understand appropriateness, future research could include a systematic and detailed assessment of the context, combined with measures of mothers’ interpretations of infant distress, the specific needs signaled by the infant (or experimentally manipulate infant needs, for instance, with established frustration paradigms), and whether and how maternal responses meet those needs.

While investigating response appropriateness is an important next step for cross-cultural developmental research, it remains of high importance to take a stance of cultural sensitivity. For instance, in our study, we documented maternal responses, such as prolonged response latency, that do not sit fully in line with Western ideal caregiving practices. Crucially, we found that this cultural variation did not seem to detrimentally impact infant distress recovery (Ugandan mothers tended to respond later but their infants recovered more quickly). Evidence that caregiving practices considered “responsive” or “appropriate” in one cultural setting may differ in another underscores the necessity of avoiding ethnocentric judgments when studying maternal sensitivity and its relation to child development across cultures. More research, ideally experimental to examine distress and the impacts of the surrounding environment, is needed to document this diversity and disentangle its effects on developmental outcomes.

Due to its observational basis, our study relied on a behavioral proxy of infant emotion regulation, using infant recovery from distress. In future research on this topic, we recommend the inclusion of noninvasive physiological measures of both caregivers and infants, to probe how variation in underlying emotional arousal and regulation may lead to behavioral differences, could further supplement these findings. This may include emerging methods such as infrared thermal imaging, which has been recently applied in cross-cultural field settings to measure infant affective arousal (Vreden et al., 2025). In concert with this would be a more detailed behavioral assessment of how infants regulate themselves (e.g., self-soothing, avoidance). Examining how this may vary cross-culturally could provide further valuable insights into commonalities and cultural differences in emotion regulation and how these are shaped by socialization experiences.

Related to emotion regulation and another direction for future studies would be to examine infant distress more closely. We coded distress intensity on a relatively broad scale, to be able to control for it in our analyses. Descriptively, in our data, distress intensity increased with age and was higher in the United Kingdom. Investigating how these age and cultural differences may have affected maternal responses and infant emotion regulation in more detail could further enhance our understanding of the interplay between the cyclical relation between infant signals, maternal behaviors, and infant regulation.

Last, another avenue for future research would be the inclusion of different caregivers. Although mothers in both samples were the primary caregivers for these infants (Holden et al., 2022), infants in Uganda were also cared for by a variety of other caregivers, including other children, while fathers played a more influential role in the United Kingdom (Holden et al., 2022). Further research is needed to understand how these other caregivers’ responses to distress might align or differ from the maternal ones presented here.

We believe that through its use of naturalistic data within a longitudinal cross-cultural design, our study contributes new insights into maternal responsiveness during infancy and offers a more nuanced understanding of how caregiving practices can shape development. However, several limitations should be acknowledged. First, while we examined response latency and style, we were unable to assess the specific cause of infant distress and therefore the fit between maternal response and infant need, thus preventing us from examining the appropriateness of responses. Second, the observational design precludes making causal inferences between specific maternal behaviors and infant recovery. In our naturalistic data, most maternal responses rarely occur on their own during a distress event: For example, a mother may attempt to soothe her infant through increased contact first and, when this fails, breastfeed the infant. In this case, was it a delayed effect of the increased contact, an effect of breastfeeding, or their combination that soothed the infant? To address this directionality requires a careful experimental design, which would represent an important complement to studying maternal responses in naturalistic and ecologically valid settings, as we have done here. Third, our naturalistic design meant that aspects of the surrounding environment could not be controlled for, such as the mother’s engagement in other household tasks (which could impact their responsiveness to their infant). However, we did not observe any instances of tasks or activities that could not feasibly be interrupted and further controlled for the mother’s distance from the infant in our analyses. Last, as our findings are based on two specific cultural contexts, they cannot be assumed to generalize to other settings with different caregiving structures or environmental conditions. However, assessing stability across diverse cultural contexts was not the goal of this research; rather, we aimed to document diversity with the goal of broadening our understanding of early emotional development.

In summary, our findings demonstrate that maternal responses to infant distress differ across cultures, shaped by cultural norms and expectations, with implications for infant emotion regulation. We found evidence of commonalities in how U.K. and Ugandan mothers responded to their distressed infant but also evidence of distinct caregiving strategies in the two settings. These differences reflect, to some extent, the broader socialization goals—autonomy in the United Kingdom and relationality in Uganda—previous research has found them to subscribe to. The shared elements of maternal responses across cultures (such as the link between response latency and infant distress recovery and how response latencies change with infant age) suggest potential universal caregiving tendencies in early infancy alongside culturally specific adaptations, which are suited to cultural norms for emotional expressiveness and regulation, as well as differences in environmental factors. Rather than taking a single model approach, with the West as the normative comparator, our study took an exploratory, context-sensitive view that avoided prescriptive judgments and instead focused on mapping patterns of caregiving within their cultural context. Our findings support the view that caregiving practices must be understood within their specific cultural contexts and that diverse, culture-specific caregiving strategies can foster healthy emotional development across cultures.

## Supplementary Material

10.1037/dev0002038.supp

## Figures and Tables

**Table 1 tbl1:** Participant Demographic Information Based on Background Surveys Conducted at the Same Time as Experimental Data Collection

Demographic information	Uganda	United Kingdom
*N* infants	41	41
Infant sex	23 female, 18 male	23 female, 18 male
Infant age at first time point (3 months)	*M*_age_ = 2.94 months (*SD* = 0.35)	*M*_age_ = 2.73 months (*SD* = 0.65)
Infant age at second time point (6 months)	*M*_age_ = 5.81 months (*SD* = 0.40)	*M*_age_ = 5.45 months (*SD* = 0.50)
*N* infants with older siblings	32	22
Maternal age at infant’s birth	15–42 years	26–41 years
Maternal ethnicity	39% Alur, 32% Lugbara, 22% other ethnolinguistic group, 7% no information available	78% White British, 12% British (not further specified), 5% White (not further specified), 2% mixed British, 2% no information available
Maternal education (highest level completed)	63% primary school education, 17% secondary education, 20% no formal schooling	12% secondary education, 88% higher education (undergraduate degree or equivalent)

**Table 2 tbl2:** Overview of Model Structures for Full Models to Examine Maternal Response Latency, Maternal Response Latency and Infant Distress Recovery, and Maternal Response Strategies

Research question	Outcome	Fixed effect: predictor variable	Fixed effect: control variable	Random effect	Model type and link function
Latency of maternal responses (Research Question 1)	Maternal response latency (s)	Infant Age^c^ × Site^d^	Maternal distance at distress onset, distress intensity, infant sex	Participant ID	Linear mixed model (Gaussian)
Latency of maternal responses and infant distress recovery (Research Question 2)	Infant recovery from distress (s)	Maternal Response Latency (s) × Site, Infant Age	Maternal distance (at distress onset), distress intensity, infant sex	Participant ID	Generalized linear mixed model (binomial), logit link function
Preferred maternal response strategies^a,b^ (Research Question 3)	Use of increased physical contact (yes, no)	Infant Age × Site	Distress intensity, infant sex, breastfeeding, engaging attention, verbal responses, nonverbal vocal responses	Participant ID	Generalized linear mixed model (binomial, logit link function)
*Note*. ID = identity.					
^a^Exemplary model structure for one of the active maternal response strategies (increasing physical contact). In total, five models with this structure were constructed to test the effect of each of the five active maternal strategies while controlling for other strategies that could co-occur with them. This means each of the five strategies featured as the outcome variable in one model and as a control variable in the remaining four models. ^b^For no active response to distress, we constructed a similar generalized linear mixed model but did not enter any other possible response strategies as control fixed effects, as by definition other strategies could not co-occur with no active response to distress. ^c^3 months, 6 months. ^d^Uganda, United Kingdom.					

**Figure 1 fig1:**
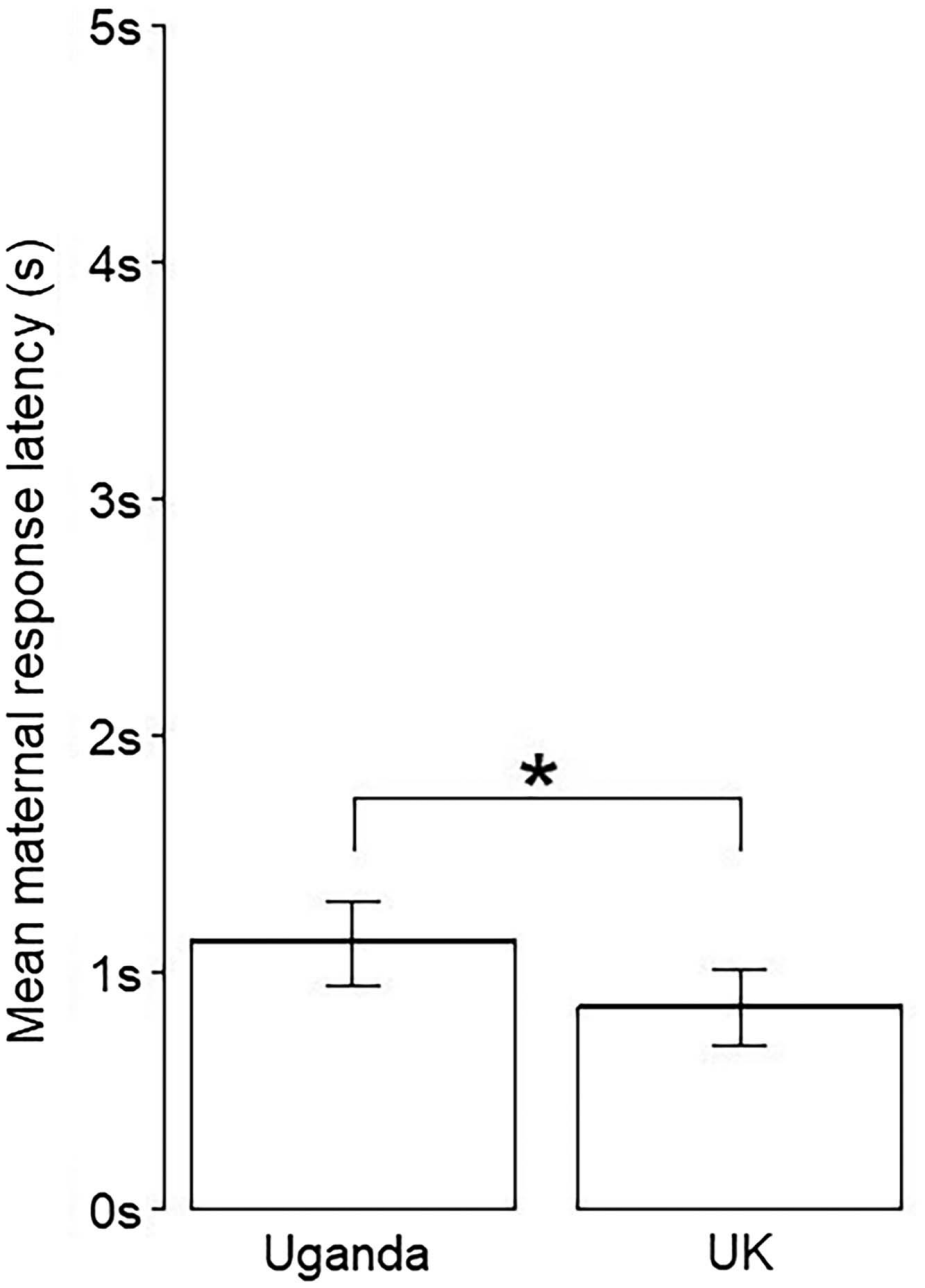
Mean Maternal Response Latency to Infant Distress by Site, Across Time Points *Note*. Bars represent means of raw data. Horizontal lines represent model estimates, and whiskers represent 95% confidence intervals around model estimates. *p < .05.

**Figure 2 fig2:**
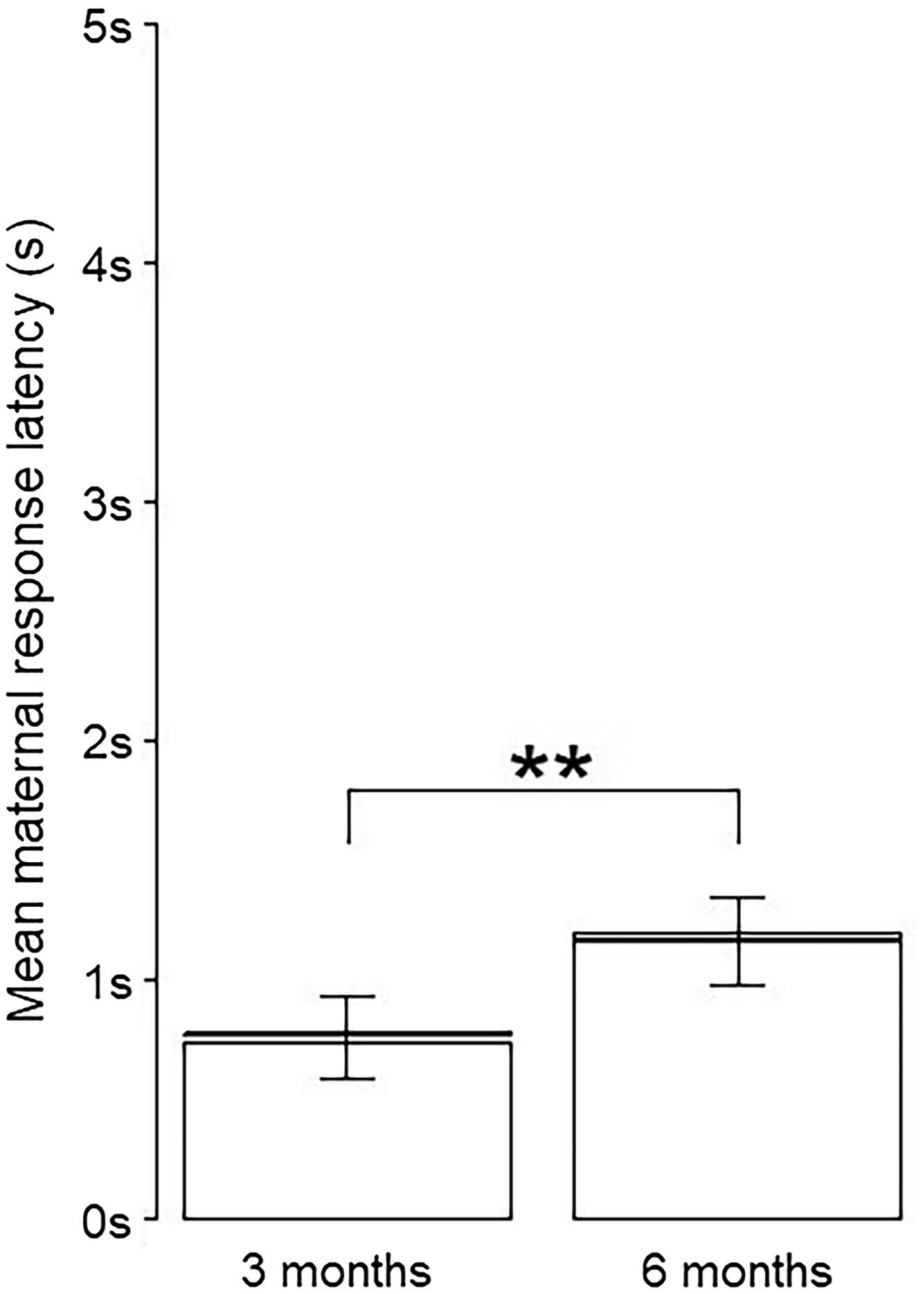
Mean Maternal Response Latency to Infant Distress by Infant Age, Across Sites *Note*. Bars represent means of raw data. Horizontal lines represent model estimates, and whiskers represent 95% confidence intervals around model estimates. **p < .01.

**Figure 3 fig3:**
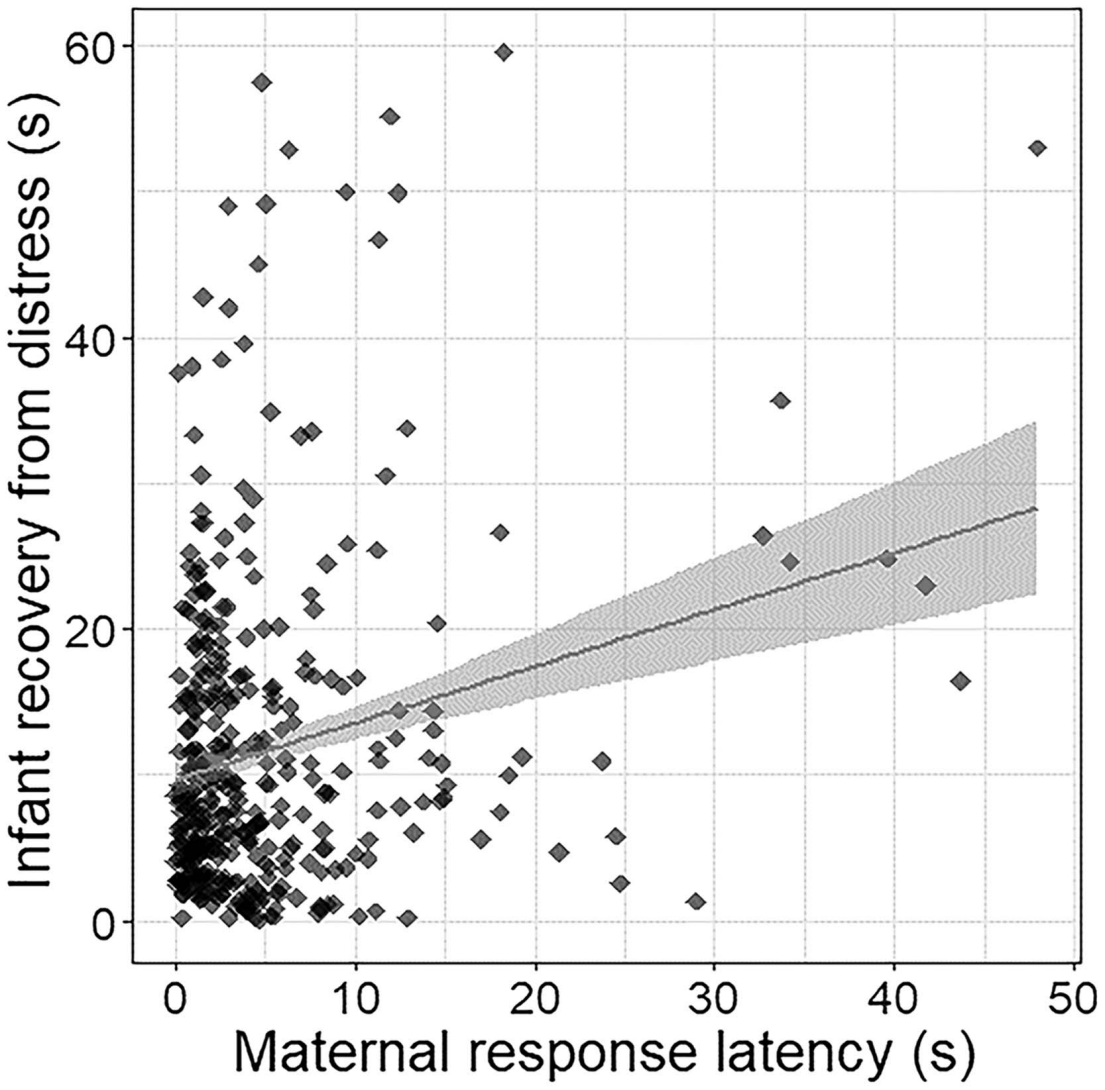
Relationship Between Maternal Response Latency and Infant Recovery From Distress *Note*. Dots represent raw data; line represents regression line with shaded confidence interval.

**Figure 4 fig4:**
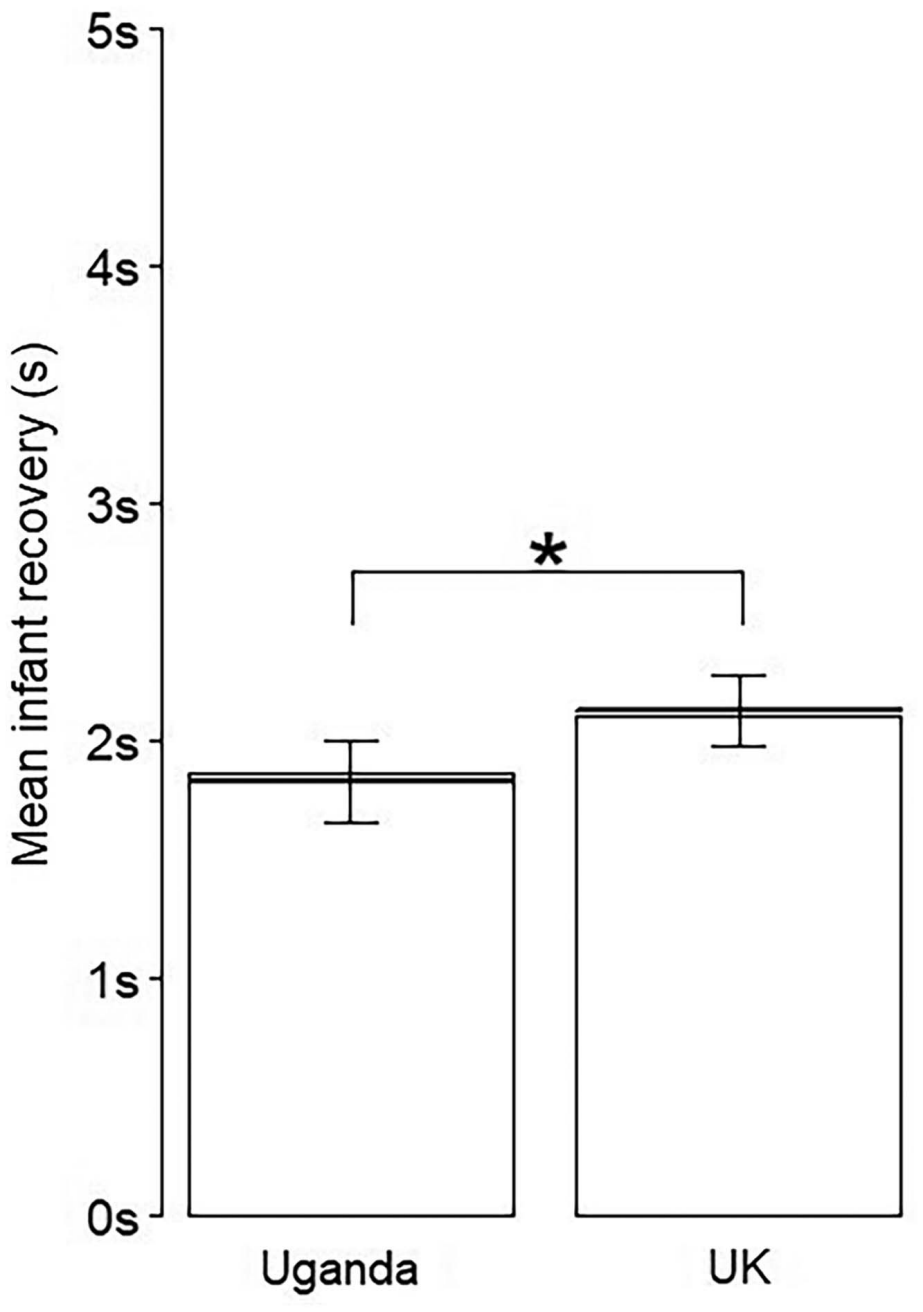
Infant Recovery From Distress by Site *Note*. Bars represent means of raw data. Horizontal lines represent model estimates, and whiskers represent 95% confidence intervals around model estimates. *p < .05.

**Figure 5 fig5:**
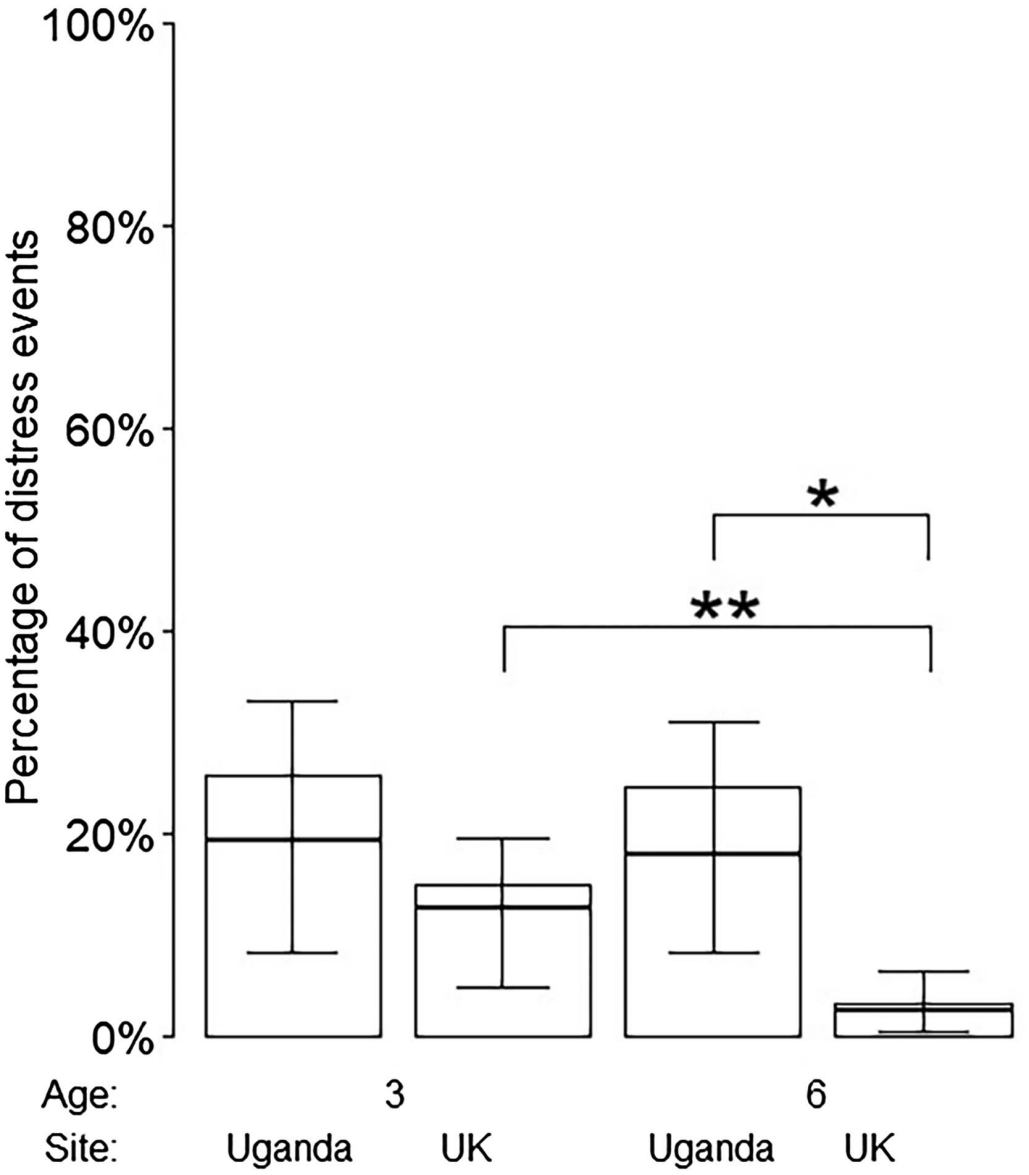
Percentage of Distress Events Which Received Breastfeeding Response by Infant Age and Site *Note*. Horizontal lines represent model estimates, and whiskers represent 95% confidence intervals around model estimates. *p < .05. **p < .01.

**Figure 6 fig6:**
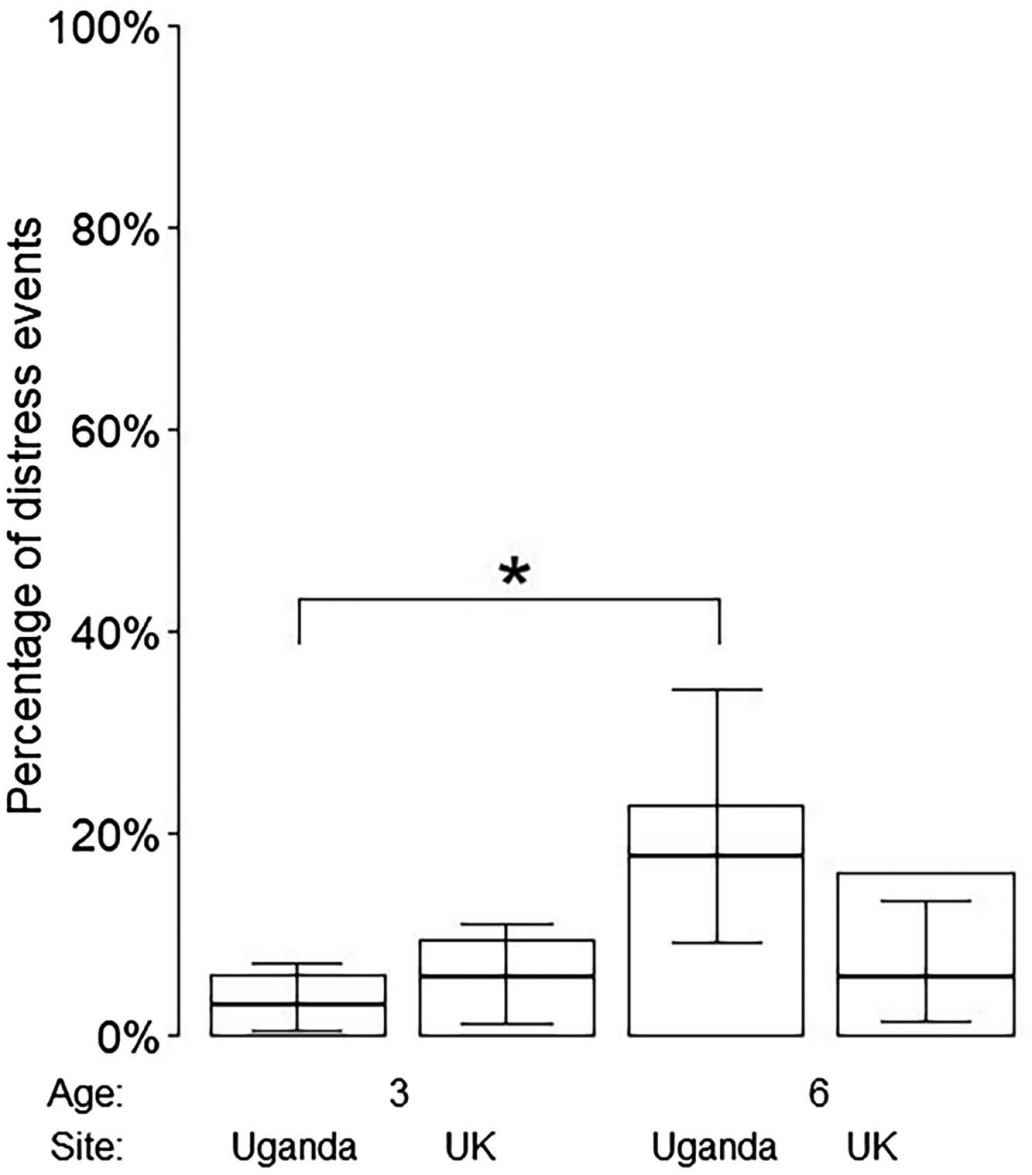
Percentage of Distress Events Which Received Engage Attention Response by Infant Age and Site *Note*. Horizontal lines represent model estimates, and whiskers represent 95% confidence intervals around model estimates. *p < .05.

**Figure 7 fig7:**
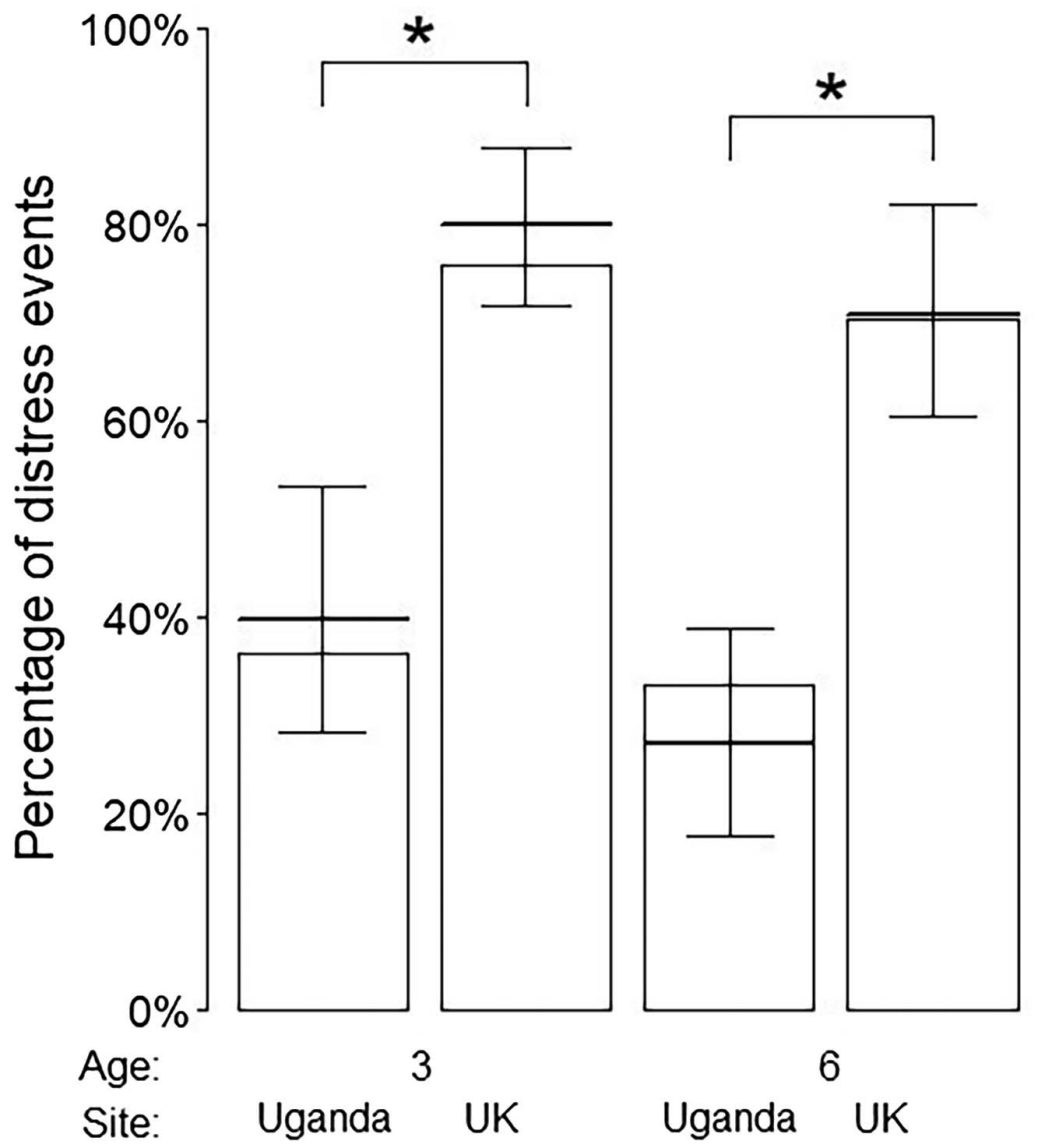
Percentage of Distress Events Which Received Verbal Response by Infant Age and Site *Note*. Horizontal lines represent model estimates, and whiskers represent 95% confidence intervals around model estimates. *p < .05.

**Figure 8 fig8:**
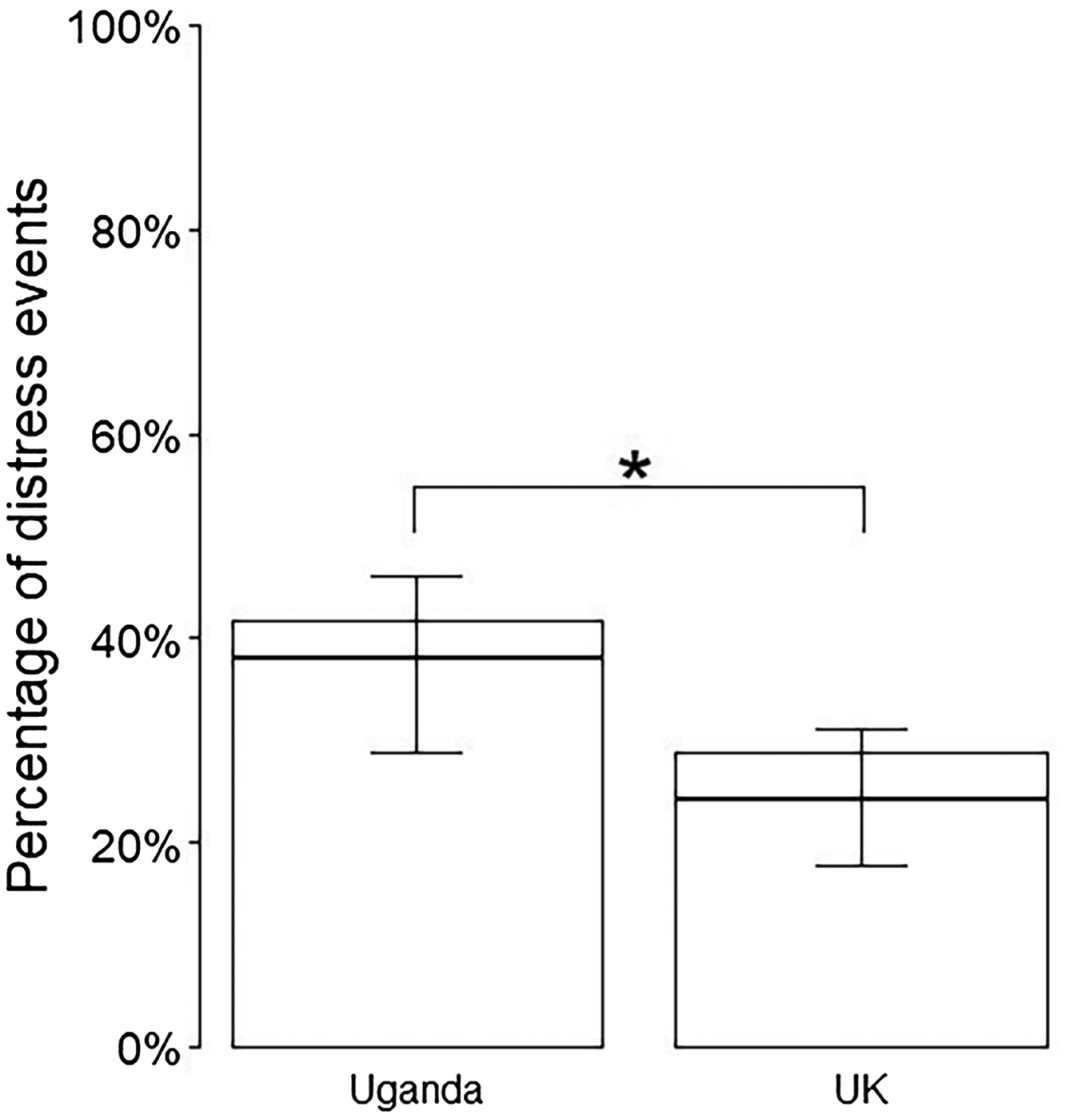
Percentage of Distress Events Which Received No Response by Site *Note*. Horizontal lines represent model estimates, and whiskers represent 95% confidence intervals around model estimates. *p < .05.

**Figure 9 fig9:**
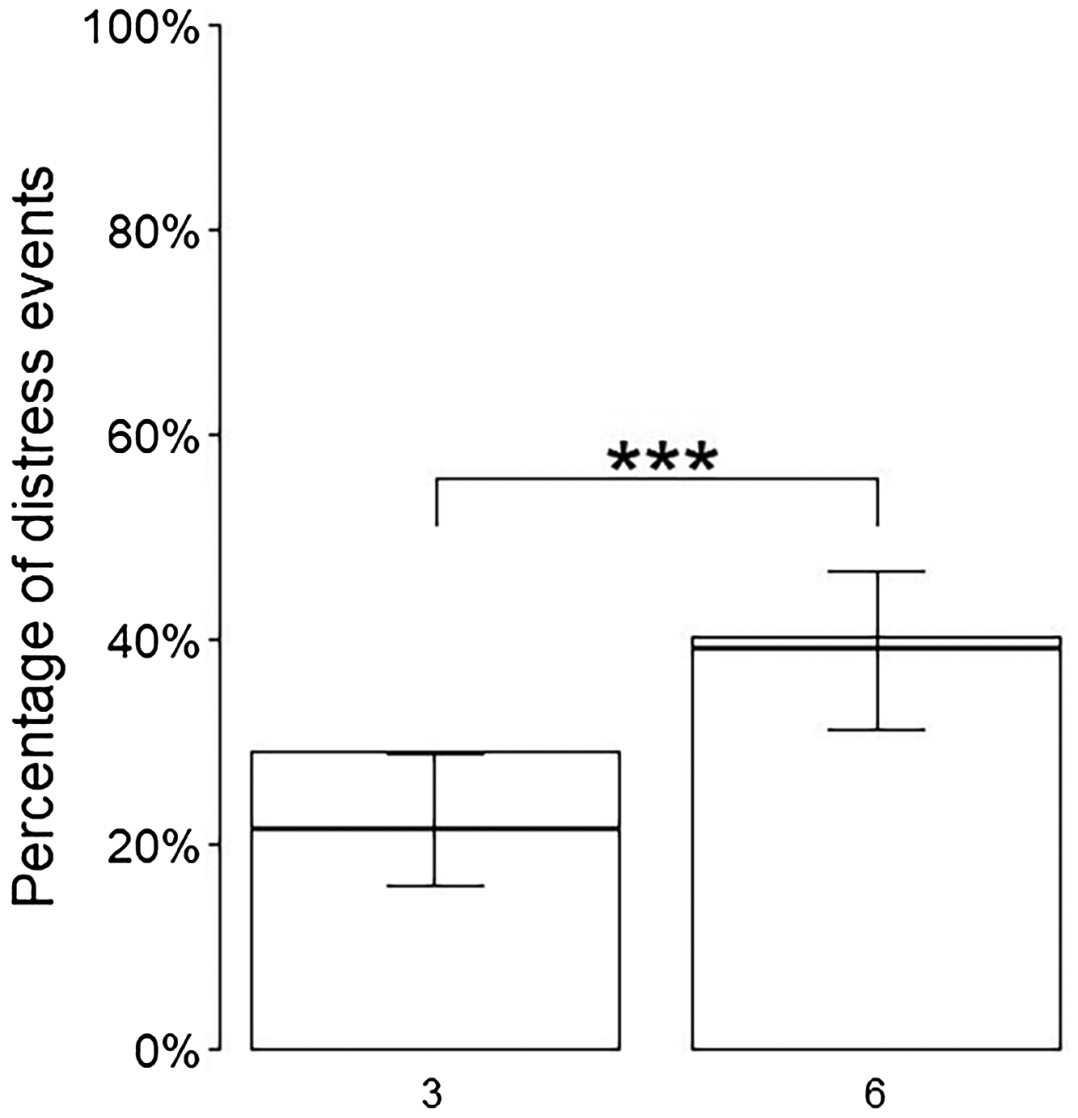
Percentage of Distress Events Which Received No Active Response by Infant Age *Note*. Horizontal lines represent model estimates, and whiskers represent 95% confidence intervals around model estimates. ***p < .001.
