# Supplementary material for: Maternal Responsiveness to Infant Distress: A Cross-Cultural Investigation in Uganda and the United Kingdom
Source: Dev Psychol. 2025 Sep 4;62(12):2591–603. doi: 10.1037/dev0002038 (PMC13738042; doi:10.1037/dev0002038)
Supplement: Supplementary file 1 [file dev0002038_Supplemental_Materials.docx]

**Supplementary Materials**

**S1 Coding Scheme**

**Table S1 Coding Scheme**

| **Infant behaviour** | | | |
| --- | --- | --- | --- |
| Category | | Definition | Coding notes |
| Distress signals | Mild-intensity distress | Mild and/or intermittent whimpering or fussing (series of low, feeble sounds), or 1 single cry sound (may be less than 2s but should be paired with other vocalisations, see coding instructions) | Distress vocalisations are considered separate events if separated by 5s of no vocalisation. Only code distress events of 2s or longer. A single cry can be less than 2s if paired with other vocalisations. |
|  | High-intensity distress | Crying (continuous, rhythmic distress vocalisation), high pitched shrieking |  |
| **Caregiver behaviour** | | | |
| Category | | Definition | Coding notes |
| No overt response |  | No change in maternal behaviour from distress onset until offset |  |
| Increase |  | Increase/add contact, initiate contact with infant or different form of contact that does not co-occur with feeding | This includes any changes in amount of physical contact, i.e., if mother and infant are already in contact, a change such as bringing the infant closer or starting to rock them would also be considered increase. |
| Vocalisation | Nonverbal | Nonverbal sounds directed towards infant, including cooing, “tsk-tsk”, humming, melodic sounds/nonverbal singing | If mother first uses nonverbal, then verbal or vice versa (e.g., humming followed by “What’s wrong?”), code as separate responses. |
|  | Verbal | Any speech directed towards infant, incl. singing, single words, or the infant’s name. | If mother first uses nonverbal, then verbal or vice versa (e.g., humming followed by “What’s wrong?”), code as separate responses. |
| Feeding | Breastfeeding | Infant is being breastfed | Also includes bringing the infant to the breast, even if the infant refuses to nurse. |
|  | Other feeding | Feeding the infant excluding breastfeeding, e.g., bottle, solids | Also includes bringing the food to the mouth, even if the infant refuses to eat. |
| Engage attention | | Engage infant’s attention, such as clapping, snapping fingers, giving infant an object, without simultaneous increase in contact | Possible for increase contact to occur before/after but engage attention itself should not be achieved through increased contact. |
| **Distance** | | | |
| Category | | Definition | Coding notes |
| In contact | | Mother and infant are touching at distress onset. |  |
| Within reach | | Mother and infant are not touching at distress onset, but the mother could touch the infant from where she is currently sitting/standing by reaching. | Mother may have to reach and lean over to touch infant, this is still considered within reach, if she can do it without taking any steps. |
| Three steps | | Mother and infant are not touching at distress onset, but the mother could touch the infant if she walked up to three steps. |  |
| Further | | Mother and infant are not touching at distress onset and the mother would have to walk more than three steps to touch the infant. |  |
| Out of shot | | Mother is not visible in the video. |  |

**S2 Distress Frequency, Duration, and Intensity**

The table below gives an overview of the available data from each site and timepoint, including the mean and range for the number of videos per infant and the number of distress events per infant, as well as the mean distress duration in seconds.

**Table S2 Mean Number and Duration of Distress Events and Relative Frequency of High and Low Intensity Distress by Site and Infant Age**

| Site | Infant age | N distress events per infant (Mean (range)) | Distress event duration (Mean (SD)) | Relative frequency of low/high intensity distress events (%) |
| --- | --- | --- | --- | --- |
| Uganda | 3 months | 5.94 (1-18) | 15.6s (12.6) | 83% low/17% high |
|  | 6 months | 9.24 (1-34) | 17.5s (18.0) | 43% low/57% high |
| UK | 3 months | 10.8 (2-23) | 15.3s (11.2) | 85% low/15% high |
|  | 6 months | 5.6 (1-20) | 21.6s (29.4) | 74% low/26% high |

**S3 Model Estimates**

Where a reduced model without interaction terms was used to test main effects of predictors (i.e., an interaction was not significant), the model estimates of this reduced model, not the full model, are presented here. All binary variables were dummy coded, with the reference category indicated in parentheses.

**Table S3.1 Latency of Maternal Responsiveness**

*Results of the reduced model of the effects of site, age, distance, distress intensity, and infant sex on maternal response latency (estimates, together with standard errors, confidence intervals, and test results).*

| Term | Estimate | SE | Lower CI | Upper CI | *χ^2^* | df | P |
| --- | --- | --- | --- | --- | --- | --- | --- |
| (Intercept) | 1.135 | 0.815 | -0.415 | 2.800 |  |  | ^(1)^ |
| Site (UK) | -0.268 | 0.130 | -0.511 | -0.015 | 4.458 | 1 | 0.035 |
| Age (6 months) | 0.391 | 0.129 | 0.139 | 0.651 | 9.330 | 1 | 0.002 |
| Distance (contact) | -0.441 | 0.805 | -2.101 | 1.034 | 5.171 | 3 | 0.160 |
| Distance (within reach) | -0.273 | 0.804 | -1.888 | 1.225 | 5.171 | 3 | 0.160 |
| Distance (further) | 0.680 | 0.975 | -1.138 | 2.487 | 5.171 | 3 | 0.160 |
| Intensity (low) | 0.222 | 0.132 | -0.032 | 0.488 | 2.774 | 1 | 0.096 |
| Sex (male) | 0.033 | 0.127 | -0.205 | 0.292 | 0.115 | 1 | 0.734 |

^(1)^ Not indicated because of having a very limited interpretation

**Table S3.2 Relationship Between Latency of Maternal Responsiveness and Infant Recovery**

*Results of the reduced model of the effects of maternal response latency, site, age, distance, distress intensity, and infant sex on infant recovery from distress (estimates, together with standard errors, confidence intervals, and test results).*

| Term | Estimate | SE | Lower CI | Upper CI | *χ^2^* | df | P |
| --- | --- | --- | --- | --- | --- | --- | --- |
| (Intercept) | 1.490 | 0.767 | 0.029 | 3.186 |  |  | ^(1)^ |
| Latency (log-transformed) | 0.108 | 0.048 | 0.016 | 0.203 | 5.146 | 1 | 0.023 |
| Site (UK) | 0.299 | 0.122 | 0.052 | 0.536 | 6.235 | 1 | 0.013 |
| Age (6 months) | -0.169 | 0.122 | -0.402 | 0.082 | 1.952 | 1 | 0.162 |
| Distance (contact) | 0.282 | 0.757 | -1.382 | 1.666 | 2.266 | 3 | 0.519 |
| Distance (within reach) | 0.088 | 0.756 | -1.574 | 1.461 | 2.266 | 3 | 0.519 |
| Distance (further) | 0.397 | 0.917 | -1.471 | 2.079 | 2.266 | 3 | 0.519 |
| Intensity (low) | -0.025 | 0.124 | -0.251 | 0.241 | 0.044 | 1 | 0.833 |
| Sex (male) | 0.262 | 0.118 | 0.045 | 0.508 | 5.147 | 1 | 0.023 |

^(1)^ Not indicated because of having a very limited interpretation

**Table S3.3 Preferred Response Strategies: Frequency of Maternal Use of Increasing Contact**

*Results of the full model of the effects of the interaction of site and age, all other possible response types (except for showing no overt response to distress), distress intensity, and infant sex on mothers’ likelihood to increase contact in response to their child’s distress (estimates, together with standard errors, confidence intervals, and test results).*

| Term | Estimate | SE | Lower CI | Upper CI | *χ^2^* | df | P |
| --- | --- | --- | --- | --- | --- | --- | --- |
| (Intercept) | 0.749 | 0.474 | -0.138 | 1.768 |  |  | ^(1)^ |
| Site (UK) * Age (6 months) | -1.125 | 0.571 | -2.299 | 0.055 | 3.709 | 1 | 0.054 |
| Breastfeeding | -0.521 | 0.338 | -1.152 | 0.203 | 2.336 | 1 | 0.126 |
| Engaging attention | -1.016 | 0.352 | -1.733 | -0.355 | 8.359 | 1 | 0.004 |
| Verbal responses | -0.082 | 0.276 | -0.633 | 0.462 | 0.088 | 1 | 0.766 |
| Vocalisations | 0.143 | 0.269 | -0.429 | 0.701 | 0.281 | 1 | 0.596 |
| Intensity (low) | 0.107 | 0.274 | -0.483 | 0.615 | 0.150 | 1 | 0.699 |
| Sex (male) | 0.165 | 0.332 | -0.464 | 0.893 | 0.244 | 1 | 0.621 |

^(1)^ Not indicated because of having a very limited interpretation

**Table S3.4 Preferred Response Strategies: Frequency of Maternal Use of Breastfeeding**

*Results of the full model of the effects of the interaction of site and age, all other possible response types (except for showing no overt response to distress), distress intensity, and infant sex on mothers’ likelihood to breastfeed in response to their child’s distress (estimates, together with standard errors, confidence intervals, and test results).*

| Term | Estimate | SE | Lower CI | Upper CI | *χ^2^* | df | P |
| --- | --- | --- | --- | --- | --- | --- | --- |
| (Intercept) | 0.1939 | 0.545 | -0.947 | 1.379 |  |  | ^(1)^ |
| Site (UK) * Age (6 months) | -1.647 | 0.843 | -12.247 | -0.197 | 4.051 | 1 | 0.044 |
| Increase contact | -0.480 | 0.338 | -1.147 | 0.163 | 2.006 | 1 | 0.157 |
| Engaging attention | -1.662 | 0.685 | -12.777 | -0.558 | 7.956 | 1 | 0.005 |
| Verbal responses | -0.860 | 0.352 | -1.611 | -0.199 | 6.166 | 1 | 0.013 |
| Vocalisations | -0.396 | 0.349 | -1.171 | 0.275 | 1.314 | 1 | 0.252 |
| Intensity (low) | -0.565 | 0.358 | -1.283 | 0.115 | 2.514 | 1 | 0.113 |
| Sex (male) | -0.155 | 0.406 | -1.024 | 0.663 | 0.144 | 1 | 0.705 |

^(1)^ Not indicated because of having a very limited interpretation

**Table S3.5 Preferred Response Strategies: Frequency of Maternal Use of Engaging Attention**

*Results of the full model of the effects of the interaction of site and age, all other possible response types (except for showing no overt response to distress), distress intensity, and infant sex on mothers’ likelihood to engage attention in response to their child’s distress (estimates, together with standard errors, confidence intervals, and test results).*

| Term | Estimate | SE | Lower CI | Upper CI | *χ^2^* | df | P |
| --- | --- | --- | --- | --- | --- | --- | --- |
| (Intercept) | -2.841 | 0.892 | -10.144 | -1.434 |  |  | ^(1)^ |
| Site (UK) * Age (6 months) | -1.941 | 0.983 | -9.606 | -0.206 | 4.452 | 1 | 0.035 |
| Increase contact | -0.976 | 0.374 | -1.853 | -0.268 | 6.836 | 1 | 0.009 |
| Breastfeeding | -1.755 | 0.700 | -12.645 | -0.613 | 8.484 | 1 | 0.004 |
| Verbal responses | 0.768 | 0.410 | -0.043 | 1.744 | 3.660 | 1 | 0.056 |
| Vocalisations | 0.513 | 0.380 | -0.286 | 1.342 | 1.811 | 1 | 0.178 |
| Intensity (low) | -0.618 | 0.405 | -1.410 | 0.136 | 2.340 | 1 | 0.126 |
| Sex (male) | 0.338 | 0.546 | -0.899 | 1.437 | 0.384 | 1 | 0.535 |

^(1)^ Not indicated because of having a very limited interpretation

**Table S3.6 Preferred Response Strategies: Frequency of Maternal Use of Verbal Responses**

*Results of the reduced model of the effects of site, age, all other possible response types (except for showing no overt response to distress), distress intensity, and infant sex on mothers’ likelihood to respond verbally to their child’s distress (estimates, together with standard errors, confidence intervals, and test results).*

| Term | Estimate | SE | Lower CI | Upper CI | *χ^2^* | df | P |
| --- | --- | --- | --- | --- | --- | --- | --- |
| (Intercept) | 0.065 | 0.447 | -0.855 | 0.952 |  |  | ^(1)^ |
| Site (UK) | 1.839 | 0.346 | 1.227 | 2.553 | 28.566 | 1 | <.001 |
| Age (6 months) | -0.538 | 0.294 | -1.219 | 0.024 | 3.346 | 1 | 0.067 |
| Increase contact | -0.075 | 0.273 | -0.619 | 0.481 | 0.074 | 1 | 0.785 |
| Breastfeeding | -0.849 | 0.351 | -1.638 | -0.199 | 6.034 | 1 | 0.014 |
| Engage attention | 0.786 | 0.392 | 0.036 | 1.570 | 4.189 | 1 | 0.041 |
| Vocalisations | -0.644 | 0.271 | -1.138 | -0.112 | 5.781 | 1 | 0.016 |
| Intensity (low) | -0.428 | 0.288 | -1.042 | 0.142 | 2.232 | 1 | 0.135 |
| Sex (male) | 0.222 | 0.331 | -0.398 | 0.929 | 0.451 | 1 | 0.502 |

^(1)^ Not indicated because of having a very limited interpretation

**Table S3.7 Preferred Response Strategies: Frequency of Maternal Use of Vocalisations**

*Results of the full model of the effects of the interaction of site and age, all other possible response types (except for showing no overt response to distress), distress intensity, and infant sex on mothers’ likelihood to use vocalisations in response to their child’s distress (estimates, together with standard errors, confidence intervals, and test results).*

| Term | Estimate | SE | Lower CI | Upper CI | *χ^2^* | df | P |
| --- | --- | --- | --- | --- | --- | --- | --- |
| (Intercept) | -0.076 | 0.469 | -1.089 | 0.711 |  |  | ^(1)^ |
| Site (UK) * Age (6 months) | 0.561 | 0.563 | -0.599 | 1.626 | 1.003 | 1 | 0.317 |
| Increase contact | 0.152 | 0.267 | -0.372 | 0.725 | 0.323 | 1 | 0.570 |
| Breastfeeding | -0.409 | 0.344 | -1.091 | 0.216 | 1.442 | 1 | 0.230 |
| Engage attention | 0.501 | 0.352 | -0.188 | 1.208 | 1.990 | 1 | 0.158 |
| Verbal responses | -0.622 | 0.271 | -1.158 | -0.120 | 5.376 | 1 | 0.020 |
| Intensity (low) | -0.109 | 0.283 | -0.675 | 0.457 | 0.148 | 1 | 0.700 |
| Sex (male) | 0.449 | 0.314 | -0.127 | 1.122 | 2.040 | 1 | 0.1532 |

^(1)^ Not indicated because of having a very limited interpretation

**Table S3.8 Preferred Response Strategies: Frequency of Maternal Use of No Overt Response**

*Results of the reduced model of the effects of site, age, distress intensity, and infant sex on mothers’ likelihood to show no overt response to their child’s distress (estimates, together with standard errors, confidence intervals, and test results).*

| Term | Estimate | SE | Lower CI | Upper CI | *χ^2^* | df | P |
| --- | --- | --- | --- | --- | --- | --- | --- |
| (Intercept) | -1.989 | 0.371 | -2.721 | -1.347 |  |  | ^(1)^ |
| Site (UK) | -0.660 | 0.283 | -1.257 | -0.087 | 5.214 | 1 | 0.022 |
| Age (6 months) | 0.846 | 0.246 | 0.388 | 1.344 | 13.046 | 1 | <0.001 |
| Intensity (low) | 1.268 | 0.268 | 0.764 | 1.814 | 26.232 | 1 | <0.001 |
| Sex (male) | 0.189 | 0.284 | -0.342 | 0.705 | 0.447 | 1 | 0.504 |

^(1)^ Not indicated because of having a very limited interpretation
